# Reliability Of A Novel Intracardiac Electrogram Method For AV And VV Delay Optimization And Comparability To Echocardiography Procedure For Determining Optimal Conduction Delays In CRT Patients

**Published:** 2009-03-15

**Authors:** N Reinsch, C Buhr, S Huptas, T Buck, T Konorza, H Wieneke, R Erbel

**Affiliations:** West-German Heart Center, Department of Cardiology University of Duisburg-Essen, Germany

**Keywords:** AV And VV Delay Optimization, Echocardiography, Intracardiac Electrogram

## Abstract

**Background:**

Echocardiography is widely used to optimize CRT programming. A novel intracardiac electrogram method (IEGM) was recently developed as an automated programmer-based method, designed to calculate optimal atrioventricular (AV) and interventricular (VV) delays and provide optimized delay values as an alternative to standard echocardiographic assessment.

**Objective:**

This study was aimed at determining the reliability of this new method. Furthermore the comparability of IEGM to existing echocardiographic parameters for determining optimal conduction delays was verified.

**Methods:**

Eleven patients (age 62.9± 8.7; 81% male; 73% ischemic), previously implanted with a cardiac resynchronisation therapy defibrillator (CRT-D) underwent both echocardiographic and IEGM-based delay optimization.

**Results:**

Applying the IEGM method, concordance of three consecutively performed measurements was found in 3 (27%) patients for AV delay and in 5 (45%) patients for VV delay. Intra-individual variation between three measurements as assessed by the IEGM technique was up to 20 ms (AV: n=6; VV: n=4). E-wave, diastolic filling time and septal-to-lateral wall motion delay emerged as significantly different between the echo and IEGM optimization techniques (p < 0.05). The final AV delay setting was significantly different between both methods (echo: 126.4 ± 29.4 ms, IEGM: 183.6 ± 16.3 ms; p < 0.001; correlation: R = 0.573, p = 0.066). VV delay showed significant differences for optimized delays (echo: 46.4 ± 23.8 ms, IEGM: 10.9 ± 7.0 ms; p <0.01; correlation: R = -0.278, p = 0.407).

**Conclusion:**

The automated programmer-based IEGM-based method provides a simple and safe method to perform CRT optimization. However, the reliability of this method appears to be limited. Thus, it remains difficult for the examiner to determine the optimal hemodynamic settings. Additionally, as there was no correlation between the optimal AV- and VV-delays calculated by the IEGM method and the echo optimization, the use of the IEGM method and the comparability to the echo has not been definitely clarified.

## Introduction

Cardiac resynchronisation therapy (CRT) with biventricular pacing has become an established electrophysiologic solution for patients with medically-refractory congestive heart failure (CHF) due to asynchronous cardiac contractions. Various studies have demonstrated improvement in symptoms, quality of life exercise tolerance and survival [1,2]. Despite clinical improvements in the majority of patients, up to 30% of CRT patients are non-responders [3,4]. There are several reasons for CRT failure, such as suboptimal device programming, incorrect positioning of the LV lead, or even residual intra- or interventricular dyssynchrony.

Intracardiac delay optimization of biventricular pacing devices has become an important tool to improve CRT therapy and the quality of life of non-responders. Optimization of atrioventricular (AV) and interventricular (VV) delays have been shown to influence hemodynamics [5-7]. Optimal AV timing increases the left ventricular preload by coupling atrial contraction to the beginning of ventricular systole. VV-delay optimization with sequential pacing can incrementally improve cardiac function compared with simultaneous biventricular pacing, presumably by reducing both inter- and intra-ventricular dyssynchrony.The most common, proven, and tested method for AV and VV optimization is echocardiography. However, echocardiography is time-consuming, so the time constraints and the lack of resources to perform the required measurements lead to a low frequency of timing optimization in the CRT patients. A novel intracardiac electrogram (IEGM) method was recently developed as an automated programmer-based method, designed to calculate optimal AV- and VV-delays and provide optimized delay values as an alternative to the standard echocardiographic procedure [8]. The purpose of this trial was to determine the reliability of this new method of AV and VV delay optimization since but a few such measurements have been generated. Furthermore, the comparability of IEGM to the best-known echocardiographic procedure for determining optimal conduction delays was verified.

## Material and Methods

### Patient Selection

All patients previously implanted with a St. Jude medical cardiac resynchronization therapy defibrillator Atlas HF (CRT-D) were enrolled in this study. Inclusion criteria for CRT were an ejection fraction <35%, a left ventricular end diastolic diameter > 55 mm, NYHA class III or IV despite optimal medical therapy, and a QRS width >140 mm. The pacing mode was DDD with a maximal tracking rate of 130 beats per minute (bpm). Optimization of AV- and VV-delay was performed about 6 month after initiation of CRT, a period after which the main beneficial effects of CRT have taken place.

Patients were excluded from the study if they (1) had no intrinsic atrial activity (atrial rate <40 bpm), (2) had atrial fibrillation at the time of study testing, or (3) were unable to provide analyzable echocardiographic images (e.g. due to an inadequate acoustic window).

### Study Protocol

All measurements took place during the morning hours. First, a routine follow-up of the CRT device was conducted. Then, the IEGM evaluation was performed using the automated programmer optimization algorithm [8]. Three consecutive measurements were performed. The heart rate was stable at ± 5 bpm during the IEGM method of AV- and VV- delay optimization. In the case of variations between the serial measurements, the three consecutive cycles were averaged for final programming.

Finally, all patients were tested with the echo-guided-optimization as described below. An echo was performed directly after the IEGM evaluation. All echocardiographic measurements were obtained by a second, independent observer.

#### Echocardiographic AV and VV delay optimization method

AV-delay was optimized using the Doppler mitral inflow method. In this method, the AV delay that optimized the timing of mitral valve closure to occur simultaneously with the onset of left ventricular systole was calculated from pulsed Doppler mitral waveforms. The VV delay associated with the highest aortic time velocity (aortic VTI) integral was considered optimal. The heart rate was stable at ± 5 bpm during echo-guided-optimization of AV- and VV-delay.

 Quantitative measurements were performed according to standard criteria published by the American Society of Echocardiography [9]. The ultrasound system used was a VIVID 7 (GE Medical Systems, Milwaukee, WI, USA). The ejection fraction (LV-EF), and the left ventricular end diastolic and systolic volumes (LVEDV and LVESV) were calculated according to the biplane modified Simpson's rule [10]. The left ventricular internal diameter in diastole (LVIDd) and systole (LVIDs) were measured in the parasternal long axis view using the M-mode. The degree of mitral regurgitation (MR) was assessed according to the American Society of Echocardiography guidelines in orthogonal apical echocardiographic images as the average of the maximal areas of the colour flow Doppler regurgitant jet within the left atrium, and also as the ratio of the regurgitant jet area to the left atrial area [11]. Pulsed Doppler velocity signals of transmitral flow were recorded at 100 mm/s with the sample volume at the tips of the mitral valve leaflets. Peak velocities were measured during rapid LV filling (E-wave) and atrial contraction (A-wave), and the velocity ratio (E/A) was calculated. Tissue Doppler imaging (TDI) of the septal-to-lateral wall motion delay (SLWMD) was performed. The technique of using tissue Doppler imaging has been recently described [12]. The left ventricular pre-ejection period (LVPEP) was calculated using Doppler aortic flow. The right ventricular pre-ejection period was calculated using Doppler pulmonary flow. The systemic cardiac output was calculated measuring the velocity time integral (VTI) across aortic Doppler flow.

### Statistical analysis

Continuous variables are given as the mean ± S.E.M. The paired t-test was used to compare echocardiographic measurements. The parameters compared were the LV-EF, LVEDV, LVESV, LVIDd, LVIDs, degree of MR, E-wave and A-wave. The Mann-Whitney-U test was used to compare the E/A ratio and the ∆ LVPEP - RVPEP. A measurement of the linear association between two variables was evaluated using the Pearson correlation coefficient. A p < 0.05 was considered statistically significant for all tests.

## Results

### Patient Population

Eleven patients with severe heart failure were enrolled in this study. Table 1 presents the clinical characteristics obtained at the time of examination of all patients. The study population consisted of 81% males, with a mean age of 62.9 ± 8.7 years. Nine (81%) patients had complete left bundle brunch block and 2 patients (19%) had complete right bundle brunch block. The mean QRS duration was 160.4 ± 29.6 milliseconds (ms). Eight (73%) patients had known coronary heart disease (CHD) and 3 patients (27%) had dilated cardiomyopathy (DCM). No adverse events were reported during the study.The left ventricular ejection fraction, as assessed by the Simpson biplane method, was 30.3 ± 9.0%. The left ventricular enddiastolic diameter was 73.5 ± 12.7 millimeters (mm) and the left ventricular endsystolic diameter was 59.4 ± 14.2 mm. The LVEDV was 239.1 ± 76.4 milliliters (ml) and the LVESV was 170.4 ± 64.1 ml. The stroke volume was assessed as 69.5 ± 20.6 ml.

### IEGM method

Table 2 shows the proposed AV- and VV-delays with the goal of maximizing hemodynamic performance. The differences of each measurement for AV- and VV-delay and the final setting are presented for each patient. Concordance for AV-delay in all consecutive measurements was found in 3 (27%) patients. The maximum difference of the proposed optimized AV-delay was 20 ms between the three measurements (n = 6 patients) and 10 ms in the remaining 2 patients.

The VV-delay was equal in 5 (45%) patients. The maximum difference of the proposed optimized VV-delay was 20 ms between the three measurements (n = 4 patients) and 10 ms in the remaining 2 patients.

### Echocardiographic values of IEGM optimization and echocardiographic delay optimization

Table 3 summarizes the echocardiographic parameters as the mean ± SD of optimized AV- and VV-delay values for the IEGM method and echocardiographic measurements.The velocity of the E-wave as a marker of LV filling was significantly different between the echocardiographic- and IEGM-methods (0.85 ± 0.27 vs. 0.75 ± 0.25 cm/sec; p < 0.05). There was no significant difference in the A-wave, and no significant difference was observed in the E/A ratio (1.58 ± 0.93 vs. 1.78± 1.69; p = ns). Significant mitral regurgitation was present in all patients and was graded as mild in 6 patients, moderate in 2 patients, and severe in 3 patients. There was no significant difference between the optimization methods. Aortic VTI did not differ significantly between the methods, albeit a slight advance was shown for echocardiographic optimization (28.5 ± 9.6 vs. 27.0 ± 8.1 cm; p = ns).The diastolic filling time as assessed by mitral inflow was significantly different between echocardiographic- and IEGM-derived optimization (492.5 ± 129.9 vs. 431.2 vs. 107.8 ms; p < 0.0001). Furthermore, the TDI of SLWMD differed significantly between both methods (37.9 ± 26 vs. 57.9 ± 34.7 ms; p < 0.05). The LV-PEP, RV-PEP and ∆ LVPEP - RVPEP did not reveal any significant differences with respect to the optimization method.

### Comparison of optimized delay values of echocardiography vs. IEGM

The optimal echo AV delay was 126.4 ± 29.4 ms and the optimal IEGM AV delay was 183.6 ± 16.3 ms (p < 0.001). There was no correlation between optimal echo and IEGM delays (R = 0.573; p = 0.066). The optimal VV delays showed significant differences as well for optimized echo delays (46.4 ± 23.8 ms) vs. IEGM delays (10.9 ± 7.0 ms; p <0.01). There was no correlation between optimal echo and IEGM delays (R = -0.278; p = 0.407). In 7 of the 11 patients (64%), RV pacing preceding LV pacing was optimal using the IEGM (Table 2) and echo methods, respectively.

## Discussion

The aim of this study was to determine the reliability of the IEGM method of AV- and VV-delay optimization, as well as the comparability to the echocardiographic procedure for determining optimal conduction delays. Our results have demonstrated that though simple and safe to perform, (1) the IEGM method displays a significant variability between consecutively performed measurements and (2) there was no correlation between the optimal AV- and VV-delays calculated by the IEGM method and the echo-derived optimization, (3) resulting in different hemodynamic parameters, mainly of the diastolic function.

### IEGM-based delay optimization

The automated programmer-based IEGM method is a quick, safe, and easy tool for the optimization of AV- and VV-delays in patients with CRT devices [8]. However, these criteria do not reflect the reliability of this method for the optimization of the cardiac activation pattern. Our results demonstrate a variability of the IEGM method of up to 20 ms in 3 consecutively performed serial optimization procedures both for AV- as well as the VV-delays. The reliability of all consecutively performed measurements was observed in less then one-half of the patients concerning both AV- and VV-delay optimization. This is of major interest as simultaneous biventricular pacing improves cardiac performance compared with the native rhythm, and hemodynamics can be further improved by individually programming both AV- and VV-delay [13-15]. As previoulsy published, pre-excitation by 20ms of 1 chamber has been shown to influence hemodynamics significantly [16]. This clearly demonstrates that the differences in delays occurring in our patients may significantly limit the benefit of CRT by means of suboptimal delay settings.

Due to a lack of chronic data on the difficulties of the IEGM-based delay optimization method, there is currently no further information regarding the expected variability or measurement-influencing factors of optimized delays in our patients that might explain these differences in measurements. Previously published, temporal variations of echocardiography-based Doppler- or two-dimensional parameters and echo-guided optimized atrioventricular and interventricular delays during follow-up have been described, too [17-18]. Nevertheless, the intra-examination variability of consecutive established IEGM delays in this study appear to be superior, thoroughly influencing the optimal clinical benefit significantly. Certainly, randomized double-blinded controlled and multicenter studies are needed to determine this method and to identify limitations that might result using the IEGM method.

### Comparison of the IEGM method with echocardiography

The results of our study showed that there was no correlation between the optimal AV and VV interval settings of these methods. These data correspond significantly with the findings of van Gelder et al. [19], who also showed no correlation between the optimal settings of the VV interval from the IEGM method and the invasive LVdp/dt measurements. However, our study contrasts the results of Becker et al. [8], who reported an optimal VV interval of 15±44 ms for the echocardiographic optimization and 13±20 ms for the IEGM method, as well as an optimal AV delay of 165±28 ms for the echocardiographic optimization and 178±16 ms for the IEGM method. Van Gelder et al. [19] assumed that the remarkably short VV delay determined by the IEGM method might be explained because of a different lead position of the left and right ventricular leads.

Additionally, we have reported that in the echo optimization there was a significant progressive lengthening of the LV diastolic filling time (DFT) and transmitral velocity during early diastolic filling (E-wave). This is of interest as these parameters may reflect improvements of diastolic function. The fact that CRT improves diastolic dysfunction itself has been previously demonstrated [20-22]. Thus, in the study of Waggoner et al. [23], pulsed-wave Doppler (PWD) mitral E-wave velocity decreased and E-wave duration and DFT increased significantly immediately after CRT. Moreover, CRT was shown to enhance diastolic filling patterns in both responder and nonresponder patients related to an improvement in symptoms [24]. However, as suboptimal pacemaker programming post-CRT may be a determinant for lack of optimal benefit, optimization of AV-delay in addition to CRT may lead to a further increase of myocardial function [13,25].  In a recent analysis by Waggoner et al. [23,26], it was shown that in heart failure patients receiving CRT, improvement in LV diastolic function is coupled to improvement in LV systolic function.

We also showed the SLWMD was significantly different between the echo- and IEGM-based optimization. This is of interest, because the different SWLMD might result in divergent VV-delays of the echo and the IEGM methods. Consequently, as a marker of intraventricular dyssynchrony, decreased SLWMD indicates a reduction of dyssynchrony and enhanced response to CRT [27]. Thus, in a previous study, SLWMD decreased significantly in responders compared to non-responders. This led to improvement in the 6-min walk test, ejection fraction and a further decrease of left ventricular end-diastolic and end-systolic diameters in the responder group [28]. Recently, acute hemodynamic studies have demonstrated an enhanced response to CRT with additional improvements in left ventricular synchrony and left ventricular function due to optimization of the VV interval [27,29]. Sequential biventricular pacing with the VV-delay optimized enhances the response to CRT compared to simultaneous CRT as it improves systolic function and reduces mitral regurgitation and LV volumes in patients with heart failure and electromechanical delay [30]. VV optimization has been shown to improve NYHA class and LVEF at follow-up [31].

## Study limitations

The number of patients was small. This study might delineate issues which should be verified in additional studies. Certainly, randomized double-blinded controlled and multicenter studies should follow this report. Furthermore, it has been shown that exercise can have a significant influence on ventricular dyssynchrony in heart failure patients [32]. For this study, all data were collected in a resting state. However, this may not reflect the hemodynamic effects when the patients are active and differences in the IEGM and echocardiographic methods may vary.The best method for adjusting the AV and VV delay to maximise longterm clinical responses is not known yet. While the echocardiogram optimization techniques are well established and supported by several clinical trials, the clinical utility of the IEGM method needs further clarification. The lack of clinical response measures over time was also a limitation of this study.

## Conclusion

 The automated programmer-based IEGM-based method provides a simple and safe method to perform CRT optimization. However, the reliability of this method appears to be limited. Thus, it remains difficult for the examiner to determine the optimal hemodynamic settings. Additionally, as there was no correlation between the optimal AV- and VV-delays calculated by the IEGM method and the echo optimization, the use of the IEGM method and the comparability to the echo has not been definitely clarified. Further studies are needed to elucidate the varieties of measurements of the IEGM method and the discrepancies between both methods found in the current study.

## Figures and Tables

**Table 1:**
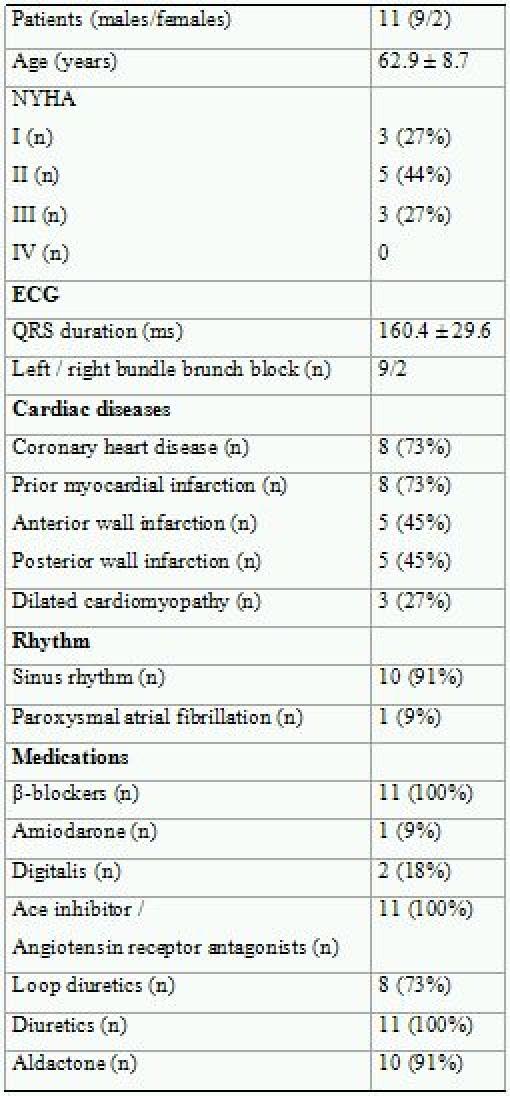
Clinical Characteristics for all patients

**Table 2:**
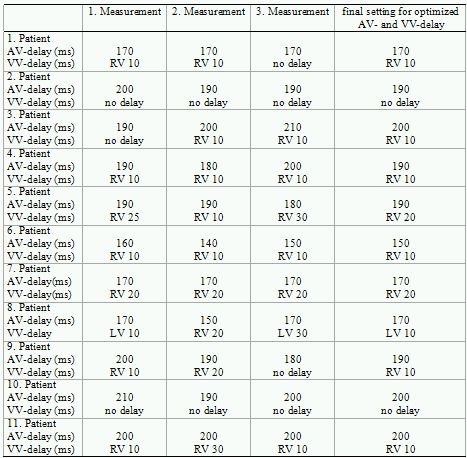
Different measurements of AV- and VV-delay using the IEGM method

RV = right ventricle; LV = left ventricle; ms = milliseconds

**Table 3:**
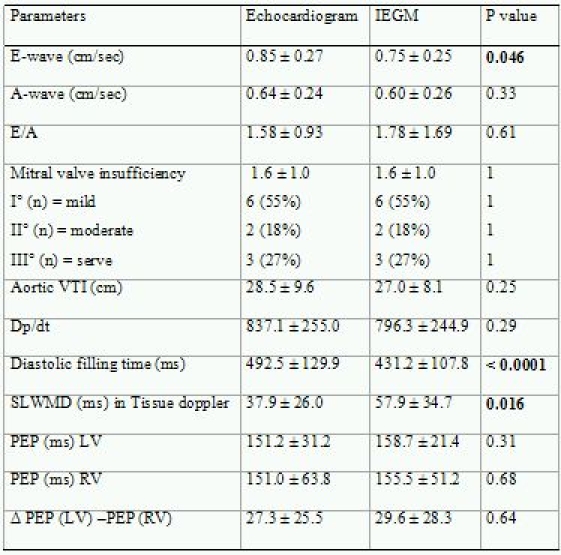
Comparison of echocardigraphic measurements in CRT patients afterAV and VV delay optimization for the IEGM- and echocardiographic methods

VTI = velocity time integral; SLWMD = septal to lateral wall motion delay;PEP = pre-ejection period
